# Free Radical Scavenging and Analgesic Activities of *Cucumis sativus* L. Fruit Extract

**DOI:** 10.4103/0975-1483.71627

**Published:** 2010

**Authors:** D Kumar, S Kumar, J Singh, Narender, Rashmi, BD Vashistha, N Singh

**Affiliations:** *Institute of Pharmaceutical Sciences, Haryana, India*; 1 *Department of Botany, Kurukshetra University, Kurukshetra-136119, Haryana, India*; 2 *Department of Botany, I.B.College, Panipat, Haryana, India*

**Keywords:** Analgesic, antioxidant, aqueous extract, *Cucumis sativus*

## Abstract

The aqueous fruit extract of *Cucumis sativus* L. was screened for free radical scavenging and analgesic activities. The extract was subjected to *in vitro* antioxidant studies at 250 and 500 μg/ml and analgesic study at the doses 250 and 500 mg/kg, respectively. The free radical scavenging was compared with ascorbic acid, BHA (Butylated hydroxyl anisole), whereas, the analgesic effect was compared with Diclofenac sodium (50 mg/kg). The *C. sativus* fruit extract showed maximum antioxidant and analgesic effect at 500 μg/ml and 500 mg/kg, respectively. The presence of flavonoids and tannins in the extract as evidenced by preliminary phytochemical screening suggests that these compounds might be responsible for free radical scavenging and analgesic effects.

## INTRODUCTION

Antioxidants are the agents that can interfere with the oxidation process by various mechanisms, such as, reacting with free radicals, chelating free catalytic metals, and acting as oxygen scavengers.[1,2] Free radicals, with unpaired electrons, are produced in normal or pathological cell metabolism and reactive oxygen species (ROS) react easily with the free radicals to convert them into radicals. Reactive oxygen species (ROS) are highly reactive molecules, that include the superoxide anion radicals (O_2_*−), hydroxyl radicals (*OH), and hydrogen peroxide (H_2_O_2_) and peroxyl radicals (ROO*).[3–6] The ROS species, as a result, generate metabolic products that attack lipids in cell membranes or DNA. Lipid peroxidation takes place in the cell membranes or the DNA involves a series of free radical chain reaction processes and is associated with several types of biological damage — DNA damage, carcinogenesis, and cellular degeneration related to aging. Cell damages are protected by their endogenous scavenging systems or by other substances.[7] Presently, the use of synthetic antioxidants has been criticized. It is usually implied that regular consumption of natural antioxidants from vegetables, fruit, tea, and herbs may contribute to a shift in balance toward an ample antioxidant status.[3] The interest in natural antioxidants, especially phytochemicals has greatly increased in recent years.[8] Many phytochemicals including phenolics, flavonoids, tannins, proanthocyanidins, and various herbal extracts have been reported as antioxidants.[9,10] Pain represents the symptom for several diseases. Analgesics only relieve pain as a symptom, having no effect on its cause.[11]

*Cucumis sativus* L. belonging to Cucurbitaceae family is commonly known as Cucumber (English), Khira (Hindi), Sakusa (Sanskrit). It is found wildly in the Himalayan regions and also cultivated throughout India. Traditionally, this plant is used for headaches; the seeds are cooling and diuretic, the fruit juice of this plant is used as a nutritive and as a demulcent in anti-acne lotions. The fruits contain an enzyme, erepsin, Vitamin B_1_ and C, ascorbic acid, proteolytic enzyme, rutin, oxidase, succinic and maleic dehydrogenases, and so on. The seeds contain α- and β-amyrin, sitosterols and cucurbitasides, whereas, the leaves contain free cucurbitasides B and C and ferredoxin. Based on its traditional use and phytoconstituents, the fruit of the plant was selected and screened for free radical scavenging and analgesic activities using *in-vitro* and *in-vivo* models, respectively.[12,13]

## MATERIAL AND METHODS

### Collection of plant materials

The fruits of *C. sativus* were collected from the local vegetable market in Kurukshetra, in November, 2008. The plant was authenticated by Dr. B.D. Vasisht, Botany Department, Kurukshetra University, Kurukshetra (Haryana, India). A voucher specimen (No. KUK/IPS/CS-1/2009) of the plant has been deposited in the Institute Of Pharmaceutical Sciences, Kurukshetra University, Kurukshetra.

### Extraction

Fresh fruits of *C. sativus* were cleaned, cut into small pieces, and macerated with water. The extract was filtered and distilled in a water bath. The extract was solidified under reduced pressure in a rotary evaporator. The yield of the extract was 8.4% w/w of fresh drug.

### Preliminary phytochemical screening

Various phytochemical methods[14,15] were used to screen the aqueous extract of *C. sativus* fruit.

### Antioxidant screening

#### The DPPH-free radical scavenging activity

Measurement of the free-radical scavenging activity of the *C. sativus* fruit extract was done by decreasing the absorbance of methanol solution of DPPH (2, 2-diphenyl-1-picryl-hydrazyl).[16] A stock solution of DPPH was prepared by dissolving 33 mg DPPH in 1 L methanol, and 5 ml of this stock solution was added to 1 ml of CS fruit extract solution at different concentrations (250 and 500 μg/ml). After 30 minutes, the absorbance was measured at 517 nm and compared with the standards of the same concentrations. The scavenging activity was calculated as the percentage of inhibition, using the following formula:

$$\%\mathrm{Antiradical}\mathrm{activity}=\frac{\mathrm{Control}\mathrm{Absorbance}-\mathrm{Sample}}{\mathrm{Control}\mathrm{Absorbance}}\times 100\mathrm{Absorbance}$$

#### Nitric oxide scavenging activity

The nitric oxide scavenging activity was measured spectrophotometrically.[17] Sodium nitroprusside (5 mM) was prepared in phosphate buffered saline and mixed with different concentrations of the extract (250 and500 μg/ml)) prepared in distilled water and incubated at 25°C for 30 min. A control was taken without the test compound but with an equivalent amount of distilled water. Then 1.5 ml of the incubated solution was diluted with 1.5 ml of Griess reagent (1% sulfanilamide, 2% phosphoric acid, and 0.1% N-1-naphthylethylenediamine dihydrochloride). The absorbance was measured at 546 nm and the percentage scavenging activity was calculated with reference to the standard.

### Animals

Albino mice (25–30 g) of either sex were selected for the experimental study. They were obtained from Haryana Agriculture University, Hisar, Haryana, India. The animals were maintained under controlled conditions of temperature (21.5 ± 2°C), humidity (60 ± 1%), and a 12-hour light / dark cycle; and were allowed free access to food (standard pellet diet) and water *ad libitum*. Albino mice (25–30 g) were divided into four groups each containing six mice. The animals were deprived of food for six hours before the commencement of the experiment, but allowed free access to water.

### Analgesic screening

#### Tail immersion test

The analgesic effect of *C. sativus* fruit extract was determined as per the reported method.[18] The tails of all the mice were marked 2 cm from the tip. The tails were immersed up to the mark in warm water kept constant at 55°C. The reaction time was determined. It was the time taken by the mice to deflect their tails. The first reading was discarded and the mean of the next three readings was recorded as the reaction time. The reaction time was recorded before and 0, 30, 60, and 90 minutes after administration of the drugs.

#### Acetic Acid-Induced Writhing Test

The analgesic activity of the *C. sativus* fruit extract was studied using the acetic acid-induced writhing model in mice. The extract doses and the vehicle were given orally 30 minutes before intraperitoneal administration of 0.7% acetic acid, and Diclofenac sodium was given intraperitonially, 15 minutes before the injection of acetic acid. After an interval of five minutes, the mice were observed for writhing (contraction of the abdominal muscles accompanied by stretching of the hind limbs) for the next 10 minutes.[19] The analgesic effect was expressed as the reduction of the number of writhings between the control and pretreated mice, and compared with the standard.

### Statistical analysis

The data was expressed as mean ± standard deviation (SD) of three readings for each experiment. The Student’s t-test was used to test the significance of differences between the results obtained for samples and controls. A probability value of less than 0.05 was considered as significant.

## RESULTS AND DISCUSSION

The aqueous extract of *C. sativus* fruit was screened for various chemical tests as per the reported methods and was found to contain glycosides, steroids, flavonoids, carbohydrates, terpenoids, and tannins [Table 1]. The free radical scavenging effects of *C. sativus* was evaluated by various *in vitro* methods, the results of which have been shown in Table 2. The DPPH radicals were used as a substrate to evaluate the free radical scavenging activities of the fruit extract. It involved the reaction of specific antioxidants with a stable free radical 2, 2-diphenyl-1-picryl-hydrazyl (DPPH*). As a result, there was a reduction of DPPH concentration by the antioxidant, which decreased the optical absorbance of DPPH; this was detected by a spectrophotometer at 517 nm. BHA and ascorbic acid were used as standards. The scavenging effect of the fruit extract of *C. sativus* on the DPPH radical was 56.15%, at a concentration of 500 μg/ml. These results indicated that the extract had a noticeable effect on scavenging the free radicals. In the nitric oxide scavenging study, a crude extract of the fruit was screened for its inhibitory effect on nitric oxide production and compared with ascorbic acid. The extract showed inhibition of concentration-dependent nitric oxide production.

**Table 1:** Preliminary phytochemical screening of aqueous extract of *C. sativus* fruits

Phytoconstituents	Aqueous extract of the *Cucumis* fruit			
Alkloids	−
Glycosides	+
Steroids	+
Flavonoids	+
Carbohydrates	+
Saponins	−
Tannins	+

+ indicates the presence and − indicates the absence of the phytoconstituent

**Table 2:** *In vitro* free radical scavenging effect of *C. sativus* fruit

Sample Tested	Sample Conc. (μg/ml)	DPPH scavenging activity (% inhibition)*	Nitric oxide scavenging activity (% inhibition)*	
CS	250	32.21 ± 1.43	39.54 ± 0.15
	500	56.15 ± 2.32	53.26 ± 2.59
Ascorbic acid	250	69.53 ± 0.64	54.64 ± 2.01
	500	90.43 ± 1.14	69.02 ± 1.41
BHA	250	70.87± 2.91	58.21 ± 0.46
	500	88.05± 1.30	72.05 ± 1.96

* Average of three determinations

The aqueous fruit extract was also screened for the analgesic effect by tail immersion and writhing effect at doses of 250 and 500 mg/kg [Tables 3 and 4]. The results were compared with diclofenac sodium taken as a standard. The extract showed significant activity at 500 mg/kg.

**Table 3:** Effects of the aqueous extract of *C. sativus* on the tail withdrawal reflex of mice induced by the tail immersion method

Groups	Dose (mg/kg)	Mean reaction time before and after treatment				% inhibition		
		0 minutes	30 minutes	60 minutes	90 minutes	30 minutes	60 minutes	90 min
Group I	-	2.64 ± 0.19	2.94 ± 1.72	2.87 ± 0.44	2.93 ± 1.35	-	-	-
Group II	250	1.96 ± 1.73	3.88 ± 0.97*	5.78 ± 0.43*	5.18 ± 0.62	24.23	50.35	43.44
Group III	500	2.27 ± 2.14	5.57 ± 0.58**	5.57 ± 0.58**	6.69 ± 0.14**	47.22	58.59	56.2
Group IV	50	2.43 ± 0.76	7.62 ± 2.43*	10.51 ± 1.13*	10.74 ± 2.57*	61.42	72.69	72.72

Group I animals received vehicle (1% Tween 80 in distilled water), Groups II and III received 250 and 500 mg/kg body weight (orally) of the crude extract of *C. sativus*, and Group IV were treated with 50 mg/kg Diclofenac sodium (i.p.). Values are mean ± SEM, (n = 6);

* *P* < 0.05

** *P* < 0.001 Student’s t- test was applied to compare with the control

**Table 4:** Effects of the aqueous extract of *C. sativus* on acetic acid-induced writhing in mice

Groups	Dose (mg/kg)	No. of wriths	% inhibition
Group I	Vehicle	32.3 ± 1.62	-
Group II	250	24 ± 0.58**	25.70
Group III	500	14.3 ± 2.41**	55.73
Group IV	50	8 ± 1.23*	75.23

Group I animals received vehicle (1% Tween 80 in distilled water), Groups II and III received CS extract 250 and 500 mg/kg body weight (p.o.) of the crude extract of *C. sativus*, and Group IV were treated with Diclofenac sodium (50 mg/kg). Values are mean ± SEM, (n = 6);

* *P* < 0.05

** *P* < 0.001 Student’s t- test was applied to compare with the control

The present study indicates that the extract has shown strong analgesic action in mice, by inhibiting the acetic acid-induced writhing and by increasing the latency period in the hot-plate test. These findings seem to, in part, justify the folkloric uses of this plant. Furthermore, it has been reported that phytochemical compounds like flavonoids and tannins, commonly found in plants have multiple biological effects, including antioxidant activity. There are also reports on the role of the flavonoid, a powerful antioxidant,[20–23] in the analgesic activity, primarily by targeting prostaglandins.[24,25] Again the plant extract demonstrated good antioxidant action in the tested models. Therefore, it can be assumed that the cyclooxygenase (COX) inhibitory activity together with the antioxidant activity may reduce the production of free arachidonic acid from the phospholipid or may inhibit the enzyme system responsible for the synthesis of prostaglandins and ultimately relieve the sensation of pain. Moreover, the reducing properties are generally associated with the presence of reductones, which have been shown to exert antioxidant action by breaking the free radical chain, by donating a hydrogen atom.[26] Therefore, antioxidants with free radical scavenging activities may have great relevance in the prevention and treatment of diseases associated with oxidants or free radicals.[27]

Upon preliminary phytochemical screening, the aqueous extract of *C. sativus* fruit was found to contain glycosides, steroids, carbohydrates, saponins, and tannins. Therefore, the presence of flavonoids and tannins in the extract suggests that these compounds might be responsible for free radical scavenging and analgesic effects of the extract. The components responsible for the antioxidative and antinociceptive activities of the fruit extract are currently unclear. Therefore, it is suggested that further studies be performed on the isolation and identification of the pure components of *C. sativus* fruits.

## References

[1] Shahidi F, Wanasundara PK (1992). Phenolic antioxidants. Crit Rev Food Sci Nutr.

[2] Sanchez MC, Larrauri JA, Saura CF (1999). Free radical scavenging capacity an inhibition of lipid oxidation of wines. Food Res Int.

[3] Halliwell B, Aeschbach R, Löliger J, Aruoma OI (1995). The characterization of antioxidants. Food Chem Toxicol.

[4] Squadriato GL, Peyor WA (1998). Oxidative chemistry of nitric oxide. The role of superoxide, peroxynitrite and carbon dioxide. Free Rad BiolMed.

[5] Yıldırım A, Mavi A, Oktay M, Kara AA, Algur OF, Bilaloglu V (2000). Comparision of antioxidant and antimicrobial activities of Tilia (*Tilia argenta* Desf Ex DC) Sage (*Salvia triloba* L.) and Black tea (*Camellia sinensis*) extracts. J Agricultural Food Chem.

[6] Gülçin I, Oktay M, Küfrevioˇglu ÖI, Aslan A (2002). Determination of antioxidant activity of *Lichen cetraria* islandica(L) Ach. J Ethnopharmacology.

[7] Halliwell B, Gutteridge JM (1990). Role of free radicals and catalytic metal ions in human disease: an overview. Methods Enzymol.

[8] Jayaprakasha GK, J Rao (2000). Phenolic constituents from lichen Parmontrema stuppeum. Hale and antioxidant activity. Zeitschrift Für Naturforscung.

[9] Xie B, Shi H, Chen Q, Ho CT (1993). Antioxidant properties of fractions and polyphenol constituents from green, long and black teas. Life Sci.

[10] Formica JV, Regelson W (1995). Review of the biology of quercetin and related bioflavonoids. Food Chem Toxicol.

[11] Tripathi KD (1999). Essentials of Medical Pharmacology.

[12] Joshi SG (2003). Medicinal Plants.

[13] Nadkarni AK, Nadkarni KM (2005). Indian Materia Medica.

[14] Khandelwal KR (2007). Practical Pharmacognosy.

[15] Kokate CK (2005). Practical Pharmacognosy.

[16] Sreejayan N, Rao MNA (1996). Free radical scavenging activity of curcuminoids. Drug Res.

[17] Govindarajan R, Rastogi S, Kumar MV (2003). Studies on antioxidant activities of *Desmodium gangeticum*. Bio Pharm Bull.

[18] Toma W, Graciosa JS, Hiruma-Lima CA, Andrade FDP, Vilegas W, Souza Brita ARM (2003). Evaluation of the analgesic and antiedematogenic activities of *Quassia amara* bark extract. J Ethnopharmacol.

[19] Ahmed F, Selim MST, Das AK, Choudhuri MSK (2004). Anti-inflammatory and antinociceptive activities of *Lippia nodiflora* Linn. Pharmazie.

[20] Brown JE, Rice-Evans CA (1998). Luteolinrich artichoke extract protects low density lipoprotein from oxidation *in vitro*.Free Rad. Res.

[21] Vinson JA, Dabbagh YA, Serry MM, Jang J (1995). Plant flavonoids, especially tea flavonols are powerful antioxidants using an *in vitro* oxidation model for heart disease. J Agric Food Chem.

[22] Gil MI, Ferreres F, Tomás-Barberán FA (1999). Effect of postharvest storage and processing on the antioxidant constituents (flavonoids and vitamin C) of fresh-cut spinach. J Agric Food Chem.

[23] Kähkönen MP, Hopia AI, Vuorela HJ, Rauha JP, Pihlaja K, Kujala TS (1999). Antioxidant activity of plant extracts containing phenolic compounds. J Agric Food Chem.

[24] Rajnarayana K, Reddy MS, Chaluvadi MR, Krishna DR (2001). Biflavonoids classification, pharmacological, biochemical effects and therapeutic potential. Ind J Pharmacol.

[25] Rao MR, Rao YM, Rao AV, Prabhkar MC, Rao CS, Muralidhar N (1998). Antinociceptive and anti-inflammatory activity of a flavonoid isolated from Caralluma attenuate. J Ethnopharmacol.

[26] Duh PD, Tu YY, Yen GC (1999). Antioxidant activity of the aqueous extract of harng Jyur (*Chrysanthemum morifolium* Ramat). Lebensmittel-Wissenschaft und Technol.

[27] Soares JR, Dinis TCP, Cunha AP, Almeida LM (1997). Antioxidant activities of some extracts of Thymus zygis. Free Rad Res.

